# A Review of Machine Learning Methods of Feature Selection and Classification for Autism Spectrum Disorder

**DOI:** 10.3390/brainsci10120949

**Published:** 2020-12-07

**Authors:** Md. Mokhlesur Rahman, Opeyemi Lateef Usman, Ravie Chandren Muniyandi, Shahnorbanun Sahran, Suziyani Mohamed, Rogayah A Razak

**Affiliations:** 1 Center for Cyber Security, Faculty of Information Science and Technology, Universiti Kebangsaan Malaysia, Bangi 43600 UKM, Selangor, Malaysia; mmarks_cse@yahoo.com (M.M.R.); p99943@siswa.ukm.edu.my (O.L.U.); 2 Center for Artificial Intelligence Technology, Faculty of Information Science and Technology, Universiti Kebangsaan Malaysia, Bangi 43600 UKM, Selangor, Malaysia; shahnorbanun@ukm.edu.my; 3 Centre of Community Education and Wellbeing, Faculty of Education, Universiti Kebangsaan Malaysia, Bangi 43600 UKM, Selangor, Malaysia; suziyani@ukm.edu.my; 4 Speech Science Programme, Center for Rehabilitation and Special Needs Studies, Faculty of Health Sciences, Universiti Kebangsaan Malaysia, Jalan Raja Muda Abdul Aziz, Kuala Lumpur 50300, Malaysia; rogayah@ukm.edu.my

**Keywords:** autism spectrum disorder, feature selection, classification, machine learning, imbalanced data

## Abstract

Autism Spectrum Disorder (ASD), according to DSM-5 in the American Psychiatric Association, is a neurodevelopmental disorder that includes deficits of social communication and social interaction with the presence of restricted and repetitive behaviors. Children with ASD have difficulties in joint attention and social reciprocity, using non-verbal and verbal behavior for communication. Due to these deficits, children with autism are often socially isolated. Researchers have emphasized the importance of early identification and early intervention to improve the level of functioning in language, communication, and well-being of children with autism. However, due to limited local assessment tools to diagnose these children, limited speech-language therapy services in rural areas, etc., these children do not get the rehabilitation they need until they get into compulsory schooling at the age of seven years old. Hence, efficient approaches towards early identification and intervention through speedy diagnostic procedures for ASD are required. In recent years, advanced technologies like machine learning have been used to analyze and investigate ASD to improve diagnostic accuracy, time, and quality without complexity. These machine learning methods include artificial neural networks, support vector machines, a priori algorithms, and decision trees, most of which have been applied to datasets connected with autism to construct predictive models. Meanwhile, the selection of features remains an essential task before developing a predictive model for ASD classification. This review mainly investigates and analyzes up-to-date studies on machine learning methods for feature selection and classification of ASD. We recommend methods to enhance machine learning’s speedy execution for processing complex data for conceptualization and implementation in ASD diagnostic research. This study can significantly benefit future research in autism using a machine learning approach for feature selection, classification, and processing imbalanced data.

## 1. Introduction

Autism Spectrum Disorder (ASD) is a neurodevelopmental disorder that causes persistent deficits in children’s social communication skills and behavior. In the Diagnostic and Statistical Manual of Mental Disorders 5 (DSM-5) [1], social communication skills are described as; (i) deficits in social-emotional reciprocity, (ii) deficits in nonverbal communicative behavior, and (iii) deficits in developing, maintaining, and understanding relationships. Furthermore, deficits in behavior are defined as restricted and repetitive patterns of behavior and fixated interest among children with autism, which is referred to as (i) stereotyped or repetitive motor movements, (ii) insistence on sameness, difficulties with changes in routine or rigid patterns of verbal or nonverbal behavior, (iii) fixated interest, and (iv) unusual response to sensory aspects. Children with sensory issues may have hyper-sensitivities or hypo-sensitivities to a wide range of senses such as sights, sounds, smells, tastes, touch, balance, and body awareness. For example, children experiencing hypersensitivity to sounds might find sounds coming from air-conditioner units as disturbing. Besides this, other issues that often relate to sensory issues are flailing of the hands or arms, walking on tiptoes, swinging back and forth, and self-harm. 

Previously, the Diagnostic and Statistical Manual of Mental Disorders 4 (DSM-IV) divided autism into four categories: (i) Asperger Syndrome, (ii) Autistic Disorder, (iii) Pervasive Developmental Disorder Not Otherwise Specified (PDD-NOS), and (iv) Childhood Disintegrative Disorder (CDD). However, these four categories have been consolidated into the Autism Spectrum Disorder (ASD) diagnosis in the Diagnostic and Statistical Manual of Mental Disorders 5 (DSM-5). DSM-5 specified ASD according to severity levels based on deficits in social communication, restricted and repetitive behavior. The severity levels consist of: Level 1—Requiring Support, Level 2—Requiring Substantial Support, and Level 3—Requiring Very Substantial Support. These are briefly discussed below.

(i). Level 1—Requiring Support: Children at this level of severity exhibit difficulties in initiating, maintaining, and engaging in social interaction activities. They also demonstrate inflexibility behavior and having difficulty in switching activities. Yet, with appropriate support, they will show an improvement in social communication skills and behavior.
(ii). Level 2—Requiring Very Substantial Support: Children at this level of severity have deficits in both verbal and non-verbal social communication skills. They face difficulties initiating social interactions and show an odd response to others in conversation or social activities. Furthermore, they have inflexibility of behavior and struggle to manage any changes in their routine activities, focus, or actions. These deficits are still noticeable even though the children are provided with appropriate support.
(iii). Level 3—Requiring Very Substantial Support: Children at this level of severity demonstrate severe deficits both in verbal and non-verbal communication skills. Due to these deficits, they show feeble effort in initiating social interaction and show minimal response to others in social interactional activities. Besides this, they also exhibit inflexibility of behavior, extreme difficulty to cope with changes in routine activities, focus, or actions.

It is essential to screen and diagnose early. Screening children with autism can be carried out by parents, teachers, and individuals without specific qualification and training. The purpose of screening and monitoring is to identify early children with developmental issues or children at-risk of language and communication difficulties. Early screening will lead to early intervention The American Academy of Pediatrics recommends that all children’s screening be done regularly to ensure that they received sufficient support to reach their full potential. There are various instruments used to screen children’s development, such as the Autism Spectrum Quotient (AQ), Social Communication Questionnaire (SCQ), and Modified Checklist for Autism in Toddler (M-CHAT). The information gathered in the screening and monitoring process can be used by a health care professional during the diagnosis process to get a clear understanding of children’s social skills or behavior.

Diagnosis can only be performed by health care professionals. In diagnosing children with autism, health care professionals will use standardized diagnostic instruments. They will also review the child’s developmental history and current behavior through an interview with parents or caregivers [2]. Currently, there are three reliable, standardized diagnostic instruments often used in diagnosing autism, namely Autism Diagnostic Observation Schedule (ADOS), Autism Diagnostic Interview-Revised (ADI-R), and Diagnostic and Statistical Manual of Mental Disorders 5 (DSM-5) [2,3].

Although clinicians use standardized diagnostic tools for ASD diagnostics, one major drawback of the method is that administering diagnostic tools require a large amount of time to conduct the assessment to interpret the scores [4]. As a solution to this problem, an intelligent method of machine learning has been proposed. The primary objective of machine learning research for ASD diagnosis is to minimize diagnostic time with improved accuracy. By reducing the diagnostic time, patients with ASD can receive immediate intervention. Another objective of the machine learning approach is to recognize the best ranked ASD features by diminishing the dimensionality of the respective input dataset. 

Machine learning is a growing field of research with the aim of constructing perfect predictive models from the respective study datasets. It encompasses search methods, artificial intelligence, mathematical modelling, and other elements of prediction. The standard machine learning approaches are neural networks, decision trees, rule-based classifiers, and support vector machines, which are automated tools that do not need much human involvement in processing data. The procedure to diagnose ASD mainly involves identifying the right class, whether ASD or non-ASD, depending on input features. The procedure may be regarded as a predictive job using intelligent methods like machine learning. As a result, to diagnose whether a child has ASD or not, specialists create automated tools or classifiers using machine learning. The automated tool is designed from the input dataset, and its efficiency is measured at how it can predict the diagnostic class by performing test instances. Software packages such as Weka, R, scikit-learn, and MATLAB toolbox have different types of machine learning algorithms embedded within them. 

This review of machine learning methods analyzes recent research for ASD diagnosis, emphasizing their significances, contributions, model performances, and limitations. This machine learning and ASD literature review also explains the process of evaluation metrics, feature selection issues, classification problems, imbalance datasets challenges, and techniques to address them. Therefore, this paper aims to review optimal feature selection for classification of ASD with the machine learning techniques to leverage the best accuracy with minimum time. In this regard, the state-of-the-art machine learning methods for feature selection and classification of ASD are critically analyzed.

This study is organized as follows: Section 2 elaborates the methodology for the review. Section 3 elucidates feature selection methods and their classification. In Section 4, we describe evaluation metrics for ASD classification. Section 5 and Section 6 present the diagnostic procedure for ASD and imbalanced datasets, including possible solutions. In Section 7, we analyze the relevant works critically using machine learning algorithms applied to ASD. A discussion is presented in Section 8. Finally, we conclude in Section 9, along with the limitations of the research and opportunities.

## 2. Methods

This literature review was conducted to analyze the use of machine learning techniques in ASD detection. The procedure followed the standards of the Preferred Reporting Items for Systematic Reviews and Meta-Analyses (PRISMA) to evaluate relevant articles [5]. The comprehensive procedure of choosing articles was based on inclusion and exclusion criteria shown in Table 1. 

We used an electronic literature search for relevant, peer-reviewed articles from Google Scholar, IEEE Xplore^®^, ScienceDirect, Scopus^®^, and PubMed databases.

The searching keywords or terms were “autism spectrum disorder,” “ASD,” “autism,” “pervasive developmental disorder,” “PDD,” “diagnosis,” “machine learning with ASD,” “mental health,” “mental illness,” “mental disorder,” “genetics,” “supervised learning,” “unsupervised learning,” “gene expression in ASD,” and “data mining.” These keywords were also used to YouTube search for clear visualization. Searches were restricted to mostly the articles published from 2015 to 2020. 

**Figure 1:**
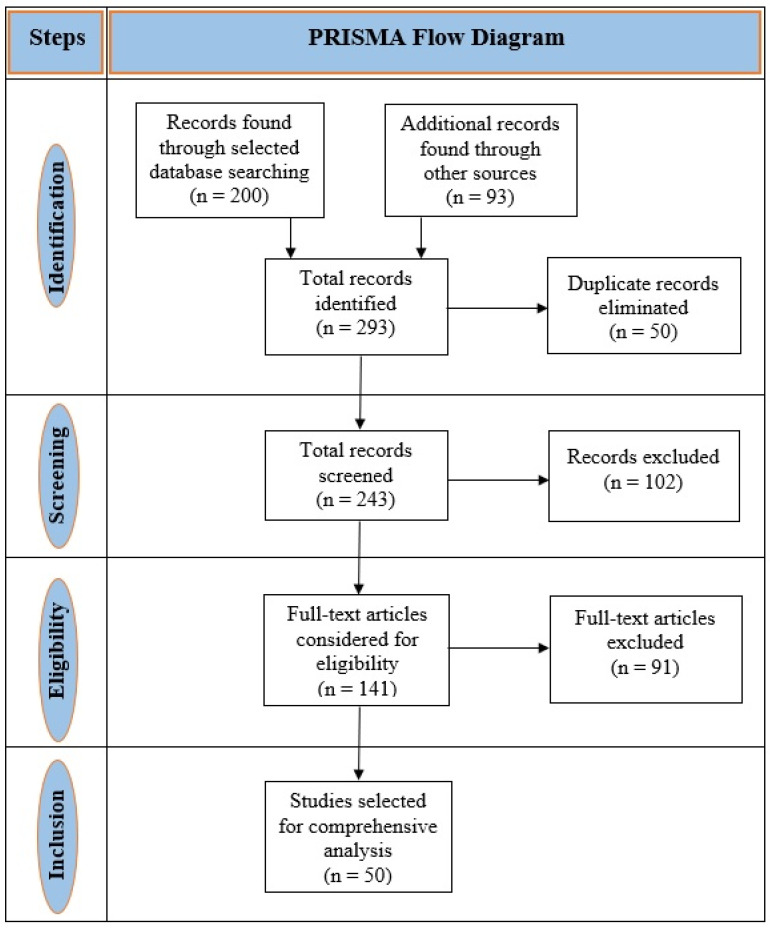
PRISMA flow diagram for searching results.

A team of six researchers who are experts in autism spectrum disorder and machine learning techniques reviewed the abstracts, methods, and results of the searched articles by maintaining the standard criteria discussed above. Finally, 50 research articles were selected after reviewing the abstracts, methods, and results. The search process and selection of the articles are shown in Figure 1. 

## 3. Feature Selection

**Figure 2:**
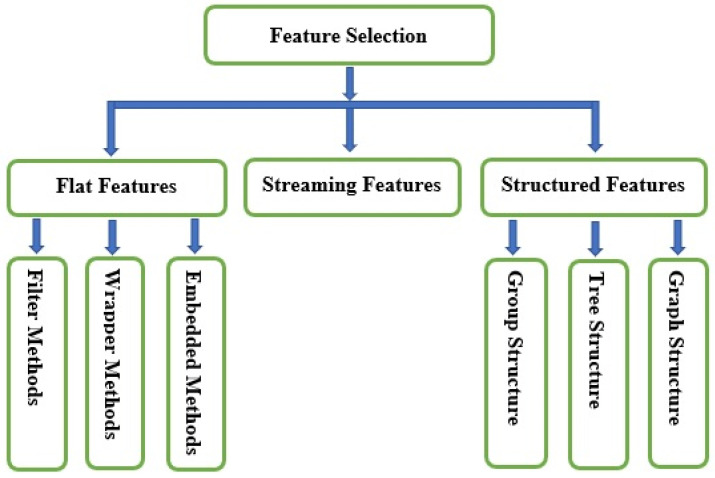
Different types of feature selection methods.

Feature selection is a machine learning technique used to decrease data dimensionality and choose relevant features to enhance classification accuracy and minimize the computational cost [6]. Figure 2 indicates the types of feature selection methods. 

Feature selection methods are categorized according to the feature structure: (i) flat features, (ii) streaming features, and (iii) structured features.

(i). Flat Features: The features are assumed to be independent. Flat feature algorithms are generally divided into two categories: filter methods and wrapper methods. Meanwhile, researchers categorize flat features’ algorithms into three types: filter methods, wrapper methods, and embedded methods [7]. Filter methods evaluate features without employing any classification algorithms. Instead, they depend on data traits, such as Pearson’s correlation and chi-square. Filter methods can choose features without classifiers. The most crucial drawback of filter methods is that they do not consider the impact of the selected feature subsets on the implementation of the training algorithm. The best possible feature subset should rely on certain biases and heuristics of the induced algorithm. Based on this fact, wrapper methods employ classifiers to classify a collection of chosen features and recommend a comfortable approach for feature selection, regardless of the type of machine learning algorithm. A typical example wrapper method is recursive feature elimination. In the embedded methods, algorithms that have inherent feature selection methods are utilized. The methods of embedding feature selection with classifier construction have the following advantages: wrapper methods include the interaction with the classification model, and filter methods are far less computationally intensive than wrapper methods; for instance, least absolute shrinkage and selection operator (LASSO) and random forest (RF), which have their own unique feature selection methods.
(ii). Streaming Features: Streaming features are features of unfamiliar sizes. They are produced dynamically and successively introduced to the classifier for prospective insertion in the model. Therefore, streaming feature selection is the selection of streaming features that impact various dynamic streaming applications. For instance, Twitter’s main blogging website generates new abbreviations (features) based on the tweets (250 million per day). Streaming feature selection is applicable in a situation where there is no need to wait for all features to be generated.
(iii). Structured Features: These features demonstrate particular fundamental structures for various real-world applications, such as disjoint or overlapping groups, trees, graphs, and temporal or spatial smoothness. Integrating knowledge about features’ structures can significantly enhance classification performance and facilitate the identification of the essential features. Structured features are divided into three categories: group, tree, and graph structures. Group structured features have many real-world practical applications. For example, various frequency bands can be presented to a model as groups in digital signal processing applications. Tree structured features represent the features that are presented in a tree-like data structure manner. Practical application of tree structured features includes image processing, where a face image’s pixels can be represented as a tree, with each parent node contains a series of child nodes. Also, genes or proteins form hierarchical tree structures during analysis, where every leaf node represents a specific feature. In graph structured features, the nodes represent the features, while the edges indicate the interactions between them.

**Figure 3:**
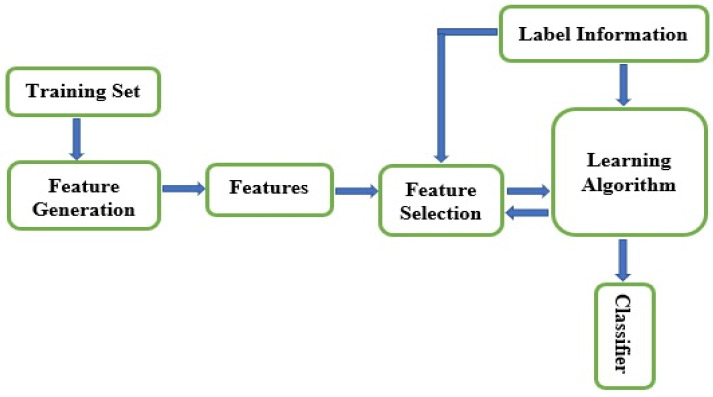
Framework of feature selection.

Figure 3 shows the general procedure for feature selection using machine learning methods. Regardless of the feature selection types and methods discussed, the goal is to reduce data’s dimensionality merely to relevant features. However, high-dimensional datasets retain irrelevant, noisy, and redundant traits. The purpose of dimensionality reduction is to obtain optimal features rapidly, since the more the data dimensionality is, the more the optimization of features will get slower. So, feature selection’s primary goal is to ascertain only minor features from a specific problem domain that signifies high-ranking classification performance [8,9].

## 4. Evaluation Metrics Utilized for Classification of ASD by Machine Learning

Evaluation metrics are used to measure the performances of machine learning predictive models. Various metrics are available for predictive models’ performance evaluation in classical machine learning. The most popular evaluation metrics for predicting ASD are discussed below.

(i). Accuracy: The accuracy of a classification problem is one of the highest universal evaluation metrics and is given in Equation (1) (TP—True Positive, TN—True Negative, FP—False Positive, TP—True Positive). The benefit of this measure is that it can find the number of test cases that have been appropriately classified from the absolute number of test cases.

(1) $$\mathrm{Accuracy}\;\left(\%\right)=\frac{\left|\;\mathrm{TP}+\mathrm{TN}\;\right|}{\left|\;\mathrm{TP}\;+\;\mathrm{TN}\;+\;\mathrm{FP}\;+\;\mathrm{FN}\;\right|\;}$$

(ii). Sensitivity (true positive rate): This metric detects the proportion of actual positives test cases that have been correctly identified. The formula for computing sensitivity is given in Equation (2).

(2) $$\mathrm{Sensitivity}\;\left(\%\right)=\frac{\left|\;\mathrm{TP}\;\right|}{\left|\;\mathrm{TP}+\mathrm{FN}\;\right|}$$

(iii). Specificity (true negative rate): This metric indicates the percentage of actual negatives test cases that have been correctly identified. The formula for computing specificity is given in Equation (3).

(3) $$\mathrm{Specificity}\;\left(\%\right)=\frac{\left|\;\mathrm{TN}\;\right|}{\left|\;\mathrm{TN}+\mathrm{FP}\;\right|}$$

(iv). Cross-validation: Cross-validation is an evaluation technique used to assess the predictive ability of a model and generate its efficiency. It is applied to the training dataset by dividing it into *k* partitions, otherwise called *k*-fold cross-validation. If the value of *k* is set to be equal to 10, then we have 10-fold cross-validation [10]. The model is trained on *k*–1 partition and examined for all partitions. By partitioning the training datasets randomly, this technique is repeated *k* times, and the average accuracy of the model is obtained from those *k* times. It is also known as stratified cross-validation since the data splitting uses random shuffling with class representation.
(v). Receiver Operating Characteristic (ROC) curve: The ROC curve, a graphical representation, is another evaluation metric used in machine learning methods to demonstrate the diagnostic capability of a binary classifier approach. Many machine learning models utilize ROC to measure their diagnostic ability [10,11,12,13,14]. The ROC curve is produced by plotting the cumulative distribution function of the true positive rate (TPR) on the *y*-axis and the false positive rate (FPR) on the *x*-axis.
(vi). Unweighted Average Recall (UAR): Another newly established evaluation metric is the unweighted average recall. This metric was utilized by Duda et al. [10] to tackle the class imbalance issue. It considers the mean sensitivity for a class and the mean of specificity for another class simultaneously without considering the number of instances.

Apart from accuracy, sensitivity, and specificity, which are the most essential evaluation metrics obtained directly from the confusion matrix, the ROC curve and UAR are also used to substantiate the efficiency of machine learning models. Moreover, when there is non-uniformity in the distributions of dataset classes under consideration, using metrics such as accuracy and ROC can be problematic. In a situation whereby a class in the datasets dominates to a very large extent, the entire data class distribution, then we have an imbalanced dataset. Therefore, to evaluate the performance of machine learning classifiers amidst imbalanced data distribution, metrics such as sensitivity, specificity, and UAR are more appropriate. 

## 5. Classification Procedure for Diagnosis of ASD

**Figure 4:**
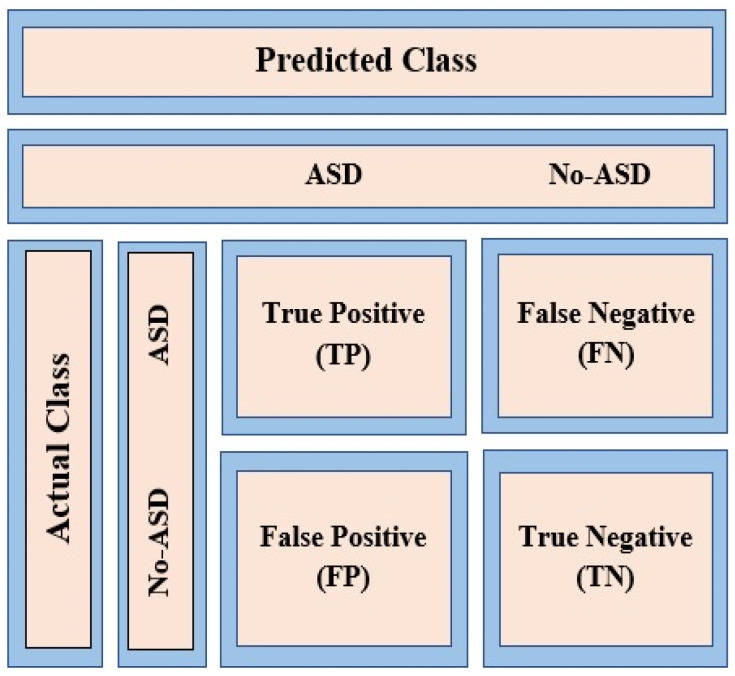
Confusion matrix for autism spectrum disorder (ASD) classification.

The primary form of the classification problem is a binary classification, for example, ASD or non-ASD, as illustrated in Figure 4. For clarity, binary classification involves grouping the entire data elements or features into two distinct classes according to already defined classification rules. This figure shows possible responses designed for a test case prediction. Previous research used machine learning algorithms for prediction purposes. A confusion matrix was utilized as a binary classification [14,15,16].

**Figure 5:**
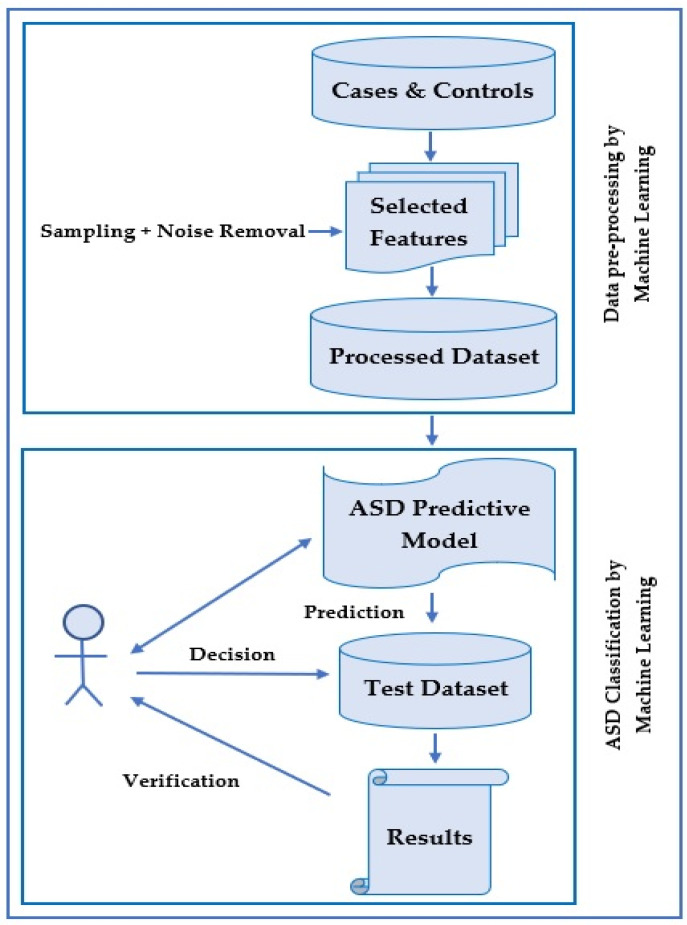
ASD classification with machine learning.

ASD diagnosis is a classification problem that consists of a few steps. The process is described in Figure 5. Here, the training dataset is input as cases and controls that have been diagnosed earlier. 

Generally, these cases and controls are created by a diagnostic tool such as ADOS-R or ADI-R in a clinic. Clinical specialists or psychologists control these tools. After identifying the training dataset, there is an optional step where feature selection is made by selecting a reduced set of features to shrink data dimensionality. 

Besides narrowing down the problem and recognizing the essential ASD features, the process of managing the computing resources is employed and incorporated during data management. A subsequent step is noise removal, which is optional. Noise can be in the form of replicated records, missing values, or imbalanced data. Recently, many studies used sampling techniques to enhance data concerns. There are two general categories of sampling techniques employed in binary classification. These are under-sampling and over-sampling techniques. In both techniques, data analysis is utilized to adjust the class distribution of a dataset. Under-sampling takes instances from the smaller class equal to those of other classes. In contrast, the over-sampling technique takes samples from a more significant class until the number of instances gets balanced. 

After completing the above preprocessing steps, machine learning algorithms are employed in the subsequent steps to classify ASD cases. Currently, software packages such as Weka and R are utilized by researchers. In order to use these software packages, related preprocessed data should be loaded. There are different options to select various filters and machine learning algorithms for data classification. Machine learning tools embedded in these types of software have more benefits in terms of having multiple evaluation metrics. Here, users can reach their expected goals, such as accuracy, sensitivity, specificity, false positive rate, false negative rate, processing rate, receiver operating characteristic (ROC), and F-measure.

The essential requirements for classifying ASD using machine learning methods are, therefore, summarized as follows:

Preprocessing stage (preliminary steps):

(i). Select features.
(ii). Dataset noise reduction and other techniques, such as treating missing values and sampling to balance the data.

Classification stage (main steps):

(i). Input: Use a specific dataset, along with controls and positive cases.
(ii). Procedure: Construct a predictive model for ASD diagnosis, with machine learning classification algorithms embedded in diagnostic tools.
(iii). Output: A predictive model that predicts the class for test data.
(iv). An authorized specialist then runs the procedure, validates the predictive model’s result, and finally decides.

## 6. Imbalanced Datasets

There are different sites where autism related datasets can be collected for research purposes. Table 2 shows various primary sources of autism datasets. However, concerning class labels, ASD datasets are imbalanced in measuring performance evaluation (such as sensitivity, specificity, error rate, area under the curve (AUC), and UAR). Most studies regarding the detection of autism have considered imbalanced datasets. However, analytical efficiency is reduced in these datasets, hence the need for more reliable and valid datasets becomes essential. 

Researchers attempt to minimize this concern by using various methods such as over-sampling, under-sampling, stratified cross-validation, and integration of datasets from multiple sources. The following are details and descriptions of the methods:

- Over-sampling: Attempts to reduce the imbalanced class label issue by reproducing the initial minority class instances (non-ASD data) are known as over-sampling. Researchers generally dislike this approach because it is time-consuming, needs high computing resources, and can overfit the training dataset. For example, Bone et al. [17] worked on balancing the class labels in the input dataset, where the ratio of ASD to non-ASD instances was 1:3. No instances were lost from the original dataset. Thus, this sampling method is commonly used in medical datasets involving imbalance difficulties.
- Under-sampling: The technique by which data instances are removed from the majority class (non-ASD) to balance the proportional dissemination of input data related to the class label is known as the under-sampling method. In this sampling method, the intention is to provide a reduced amount of information given by the predictive model, which is a critical issue for prediction and decision making because under-sampling deduces real data instances from the majority class. However, for ASD screening, early detection is essential to plan proper steps to address autism in children at an early age. Intelligent and domain-specialized sampling techniques are required to ensure reliable outcomes, reducing the deficiency of statistical deduction that simplifies the model’s performance. Over-sampling and under-sampling approaches with a preliminary clustering step seem to be a way forward, with the assumption that data duplication or data removal would be innovative more than absolutely random. Researchers categorize their input dataset into *N* clusters in the under-sampling method. The proportion of majority class instances to the minority class is used to choose instances that may be utilized for the training stage from various groups based on the computed proportion.
- Other methods of under-sampling use *k*-nearest neighbors (KNN) from supervised learning. These methods are suitable for decreasing randomization in the sampling process. In these methods, most class instances are taken from various subsets of data depending on a distance function metric.
- Stratified cross-validation: To address the problem of the imbalanced dataset, Duda et al. [10] proposed stratified cross-validation and under-sampling methods, which enabled their classification model to learn 90% and 10% features from the training and testing datasets, respectively. These methods were applied in a total of 10 different phases. They achieved a proportion of ASD to attention deficient hyperactivity disorder (ADHD) rate of 1.5:1 in every sample set. They randomly employed 10 samples in the under-sampling technique for the majority group of ASD in both training and testing sets to get this outcome.
- Integration of datasets from numerous resources: Class labels of imbalanced data are minimized, and ASD diagnostic model performance is simplified to integrate data from various resources. It has significance in removing similar features before integrating cases and controls from multiple resources. In addition, codes in the ASD predictive tools are related, such as the modules in ADOS-R. Consequently, an appropriate tactic is to remove significant similarities among features before integrating datasets. The aim is to get dissimilarities in the new integrated features so that these dissimilar features can be measured obviously as the class at the time of feature selection and diagnosis.
- Finally, regarding the evaluation metrics for constructing an ASD diagnosis model, divergence occurs when processing imbalanced data. For example, Kosmicki et al. [18] presented classifier integrity using classification accuracy as an evaluation metric. On the contrary, Duda et al. [10] argued that measuring the evaluation from imbalanced datasets is not proper. Instead, UAR is a more appropriate metric that can integrate ASD and non-ASD recall.

## 7. Review of Recent Works Based on Machine Learning

In Table 3, we have summarized the relevant works utilizing machine learning algorithms for the classification of ASD. As mentioned in Table 3, the study underscores essential issues in using machine learning to analyze and predict ASD; for example, in handling imbalanced data, multidimensional data, and appropriate feature selection for efficient classification, and most notably the competent performance of evaluation measures such as accuracy, sensitivity, specificity, ROC curve, and UAR. Machine learning enables the effective processing of complex ASD datasets in multidimensional data representing genetic information and is capable of manipulating large and abundant datasets. Therapeutic responses through signs and characteristics can also help learn ASD patterns to build a useful machine learning model by devising knowledge bases and rules for analysis and prediction. Previous studies also showed that, through machine learning models, the diagnostic time could be reduced significantly. Machine learning with adaptive feature selection in the entire feature space could also evaluate different subsets combined with impacts.

This study analyzes state-of-the-art investigations on ASD using machine learning methods. From these investigations, we identify five pivotal issues:

(i). Classifying ASD by enhanced new machine learning techniques.
(ii). Decreasing the processing time of ASD diagnosis with minimum human interruption.
(iii). Identifying the classification features that differentiate ASD and other neurodevelopmental disorders.
(iv). Finding the main features affecting ASD.
(v). Minimizing the features that contribute to current ASD methods without impeding the evaluation measures (accuracy, sensitivity, and specificity).

In the following sections, the pivotal issues are reviewed and extensively discussed. 

### 7.1. Classifying ASD by Enhanced New Machine Learning Techniques

Reaven et al. [27] used two standardized diagnostic tools referred to as gold standards, i.e., ADOS and ADI-R. The study aimed to examine the extent to which ADOS and ADI-R assessment packages can diagnose ASD. 

Supervised machine learning algorithms have also been used for ASD classification problems. An algorithm is utilized in supervised learning to predict a target variable (i.e., the dependent variable) from the input variables. This target variable is otherwise considered as a categorical or continuous variable. As an example, Hyde et al. [28] demonstrated numerous patterns and development of supervised machine learning techniques in autism. They discussed that this method allows for multidimensional data, genetic data, and large and abundant datasets.

Stevens et al. [29] identified and analyzed behavioral phenotypes in ASD using unsupervised machine learning as well. The authors mainly applied Gaussian mixture models and hierarchical clustering. This analysis aimed to evaluate the behavioral features of autism and analyze the therapeutic response through the signs and characteristics identified.

To classify ASD, Parikh et al. [30] upgraded and evaluated the performance of nine machine learning models using personal characteristic data (PCD). They improved ASD diagnosis by taking PCD as an input feature along with enhanced machine learning techniques. These models can create an additional realistic approach to diagnose ASD when combined with external attributes (e.g., functional magnetic resonance imaging (fMRI)). The analysis’s major disadvantage comes from how the ABIDE data are collected because data from 17 clinical and testing sites were obtained for this work.

Thabtah [31] explored recent clinical and screening machine-learning research in ASD. The study showed limitations of traditional methods and highlighted the essential issues that are needed to be addressed. Such issues included data, diagnosis time, and feature selection method, all related to machine learning for ASD classification. In another article, Thabtah et al. [32] also proposed a new machine learning approach known as rule-based machine learning (RML), which recognizes autistic features of positive cases and controls. The approach provides users with knowledge bases and rules, which depict the significant features under the classification. They argued that the approach showed greater predictive accuracy, specificity, harmonic mean, and sensitivity than other machine learning techniques like bagging, boosting, rule induction, and decision trees. 

Deep learning has had a growing impact on research, such as speech analysis [33] and ASD classification [20,22]. Heinsfeld et al. [20] detected ASD by applying deep learning algorithms on a large brain-imaging dataset from ABIDE (functional brain imaging data: ASD, 505; typical control, 530). For this purpose, they used 10-fold cross-validation as a sampling method. The study suggests that large multi-site datasets can be classified consistently by deep learning techniques. Compared with single-site databases, classification across several sites needs to consider additional sources of variation in subjects, scanning techniques, and facilities. This type of variation includes noise in brain imaging data that can hinder the ability to identify brain activity patterns that help distinguish disease states. Despite noise created by equipment and surroundings, the classification accuracy indicates promising results using machine learning algorithms with clinical databases and the potential use of machine learning to aid mental illness detection in the future. In this case, 70% classification accuracy was achieved, whereas Kong et al. [22] attained 90.39% accuracy. The analysis also applied deep learning algorithms on T1-w MRI data collected from ABIDE (ASD, 78; typical control, 104; average age, 15 years) and achieved 84.37% sensitivity and 95.88% specificity.

Jacob et al. [34] described how the longitudinal model and computational analytics process might boost inferential capacity to identify debilitation early that might or might not exceed formal diagnosis thresholds of ASD. The study focuses on supervised and unsupervised algorithms for detecting the neuro-developmental heterogeneity of ASD. 

Modified Checklist for Autism in Toddlers (M-CHAT) includes 23 items, while the revised M-CHAT (M-CHAT-R) has 20 items. Because of these two tools’ shortcomings, the guardians on medium-risk toddlers were applied to Modified Checklist for Autism in Toddlers-Revised/Follow-Up (M-CHAT-R/F), which included interview questions to corroborate the risk. Achenie et al. [24] applied a feedforward neural network (fNN) to M-CHAT-R/F data to enhance ASD risk diagnosis precision. The data comprised ASD features of 14,995 toddlers aged 16–30 months. High classification accuracy (not less than 99.64%) was achieved for ASD features of 14, 16, and 18 months aged toddlers. Among these, the best result was 99.95% using ASD features of 18 months old aged toddlers extracted from the females’ category of the dataset.

Another approach, such as pattern classification and stratification, has been used by Wolfers et al. [35]. The study included 57 and 19 studies on pattern classification and stratification screened respectively in 635 different studies. They found significant variation in pattern classification in the studies with predictive accuracies ranging from 60% to 98%. The reason attributed to the above includes different variety of validation methods used across studies, the variability of ASD, sampling bias, and variations in data quality, among other factors. The stratification studies were less broad, with just two replications recorded and a limited number of studies indicating external validity.

From the above analysis, machine learning methods have extensively been used in ASD studies recently. Researchers have developed predictive models for diagnosing ASD and applied machine learning techniques as the best intelligent approaches. Examples include supervised machine learning, unsupervised machine learning, and deep learning, specifically Gaussian mixture, hierarchical clustering, RML, and support vector machine, etc., with outstanding performances. Hence, machine learning approaches for diagnosing ASD are a promising field of research and can boost predictive models’ accuracy. 

### 7.2. Decreasing the Time for the ASD Diagnostic Procedure with Minimal Human Interruption

For clinical diagnosis of ASD, Allison et al. [36] showed how the pre-diagnostic time could be decreased prior to specialist assessment in the clinic to address time-consuming, lengthy, and complex process challenges characterized by the clinical diagnosis procedure for the autism diagnosis. They suggested that the medical staff (such as clinical staff, care staff, physicians, nurses) can use the top 10 questions for quick referral decisions for other ASD diagnostic cases. 

Duda et al. [10] claimed that their machine learning technique could reduce ASD diagnosis time. They argued that reducing the diagnostic time depends on the pre-diagnosis of ASD before employing machine learning methods. So, their methodology was to choose the significant features of the input data wisely so that the features could have an immediate impact on ASD classes. Advantageously, their strategy decreased input data dimensionality and the required time to build a model to minimize diagnostic time and improve efficiency.

Tariq et al. [12,37] applied machine learning to home videos of children and claimed that their method decreased the classification time of ASD. These two studies explained that recent standard techniques of diagnosing ASD take a few hours to complete because they evaluate from 20 to 100 behaviors. In an earlier study [37], they applied eight machine learning algorithms to 162 home videos (duration 2 min) for American children with and without ASD. The study aimed to assess the ability of machine learning algorithms to detect ASD unfailingly on the mobile platform and achieved the highest accuracy of 92%. In the recent study [12], the authors applied a new technique to their model with two classification layers. The first layer separated typical and atypical characteristics, while the second layer differentiated ASD and non-ASD. In this case, they also considered 159 home videos of Bangladeshi children on a mobile platform and achieved 76% accuracy in detecting atypical children from developmentally delayed children and 85% accuracy in recognizing children with ASD compared to other developmental delays. In these two cases, the authors claimed that their new technology could rapidly determine the ASD classification accurately by tagging features of home videos of children using machine learning on the mobile platform.

To accelerate ASD diagnosis, specific features should be selected carefully, which are significantly related to ASD complexities. Mythili et al. [38] suggested data mining classification systems for ASD prediction. They utilized a support vector machine, fuzzy logic, and neural network to develop the classification system. They concluded that if the classification rules can be appropriately employed on training data to create a model, the model will classify the new data accurately and timely. 

The emphasis of the machine learning approach to ASD diagnosis should not be limited to achieving a high prediction accuracy alone but also minimizing prediction time. Therefore, ASD diagnostic specialists emphasize the processing time of the classification procedures, i.e., how quickly the models yield the desired outcome while maintaining high accuracy with minimum human interruption. Hence, different researchers focus on different concerns. Examples of these concerns include pre-diagnosis time, removing abundant data, choosing only significant attributes that have an immediate impact on ASD classes, appropriately employing classification rules, and so on. 

### 7.3. Identifying the Classification Features That Differentiate ASD and Other Neurodevelopmental Disorders

To determine autism and other neurodevelopmental disorders, such as childhood-onset schizophrenia (COS), Reaven et al. [27] applied ADOS and ADI-R in the clinic. The authors categorized children into three types: A, B, and C, according to their ability to meet both ADOS and ADI-R criteria for the diagnosis of autism. They identified specific features that were very sensitive to distinguish the differences between autism and other disorders.

Auyeung et al. [11] claimed that the scores for people with autism spectrum disorder and the general public varied substantially. The ROC curve revealed excellent reliability of the test–retest and extreme internal stability; the sensitivity and specificity of AQ-Child (aged between 4 to 11 years) were notable at 95%. It consists of five areas: social skills, communication, attention switching, attention to detail, and imagination. 

Duda et al. [21] developed a unique model, mobile autism risk assessment (MARA), comprised of a 7-queries screen to differentiate ASD and other developmental disorders (DD). The authors used alternating decision tree (ADTree) in their model with 891 ASD and 75 non-ASD (16 months to 17 years old children) cases from the Autism Genetic Resource Exchange (AGRE), the Simons Simplex Collection (SSC), and the Boston Autism Consortium (Boston AC) data repositories. They achieved 89.9% sensitivity and 79.9% specificity in their analysis. Despite the model’s excellent performance, one major limitation of MARA was that it could only be utilized by only one big academic health center but not suitable for diverse medical centers. The research was undertaken during the transition of ASD requirements between a Text Revision of Diagnostic and Statistical Manual of Mental Disorders-Fourth Edition (DSM-IV-TR) and updated Diagnostic and Statistical Manual of Mental Disorders-Fifth Edition (DSM-5). Though detailed evidence was obtained for a subset of patients, it did not show any substantial difference in diagnostic results depending on the criteria of DSM-IV-TR and DSM-5. Variations in diagnostic exercise arose from the updates in criteria. In the other two works, Duda et al. [10,23] also employed machine learning techniques to differentiate ASD and attention deficit hyperactivity disorder (ADHD). The objective was to find an algorithm that could best distinguish between ASD and ADHD. They selected six machine learning algorithms [10].

In contrast, the authors [23] applied five machine learning algorithms: elastic net (ENet), LASSO (using logistic regression with *L*_1_ regularization), linear discriminant analysis (LDA), logistic regression with *L*_2_ regularization (Ridge), and support vector classification (SVC) to train on archival and survey data samples. In these two studies, the dataset for initial analysis consisted of 2925 (ASD, 2775; ADHD, 150), and the subsequent analysis consisted of 3347 (ASD, 3023; ADHD, 324). The sample completed the Social Responsiveness Scale (SRS) score sheets from the AGRE [39], the SSC [40], and the Boston AC data repositories. The two classes classified and achieved an accuracy of 96% and 89%, respectively.

Hegarty et al. [41] demonstrated that genetic and environmental impacts on the structural brain measurements contributed to ASD and TD twins. They applied T1-weighed MRI data, which were collected from children between the ages of 6 to 15 years. They proved that the structural brain elements such as surface area, brain size, cerebral and cerebellar Gray Matter (GM) and White Matter (WM) volume, cortical thickness, and mean curvature were influenced by genetic and environmental factors for the development of ASD and TD twins. They determined that for ASD twins, the cerebellar WM volume and cortical thickness were mainly instigated by the environmental factors.

In summary, specific features that discriminate ASD from other neurodevelopmental disorders are of great importance. Such disorders include ADHD, COS, ICD (Impulse Control Disorder), and TD, etc. Certain attributes affect the ASD classification procedure and can contribute significantly to ASD diagnosis and prediction accuracy. 

### 7.4. Finding the Top-Rated Features Affecting ASD

Miscellaneous results of studies on structural changes in brains with developmental disorders are available. In a systematic review conducted by Pagnozzi et al. [42], the latest (post-2007) high-resolution 3T MRI research exploring ASD-associated brain morphology was combined to recognize powerful brain imaging genetic markers. A systematic search was performed for related studies in three databases, Scopus, PubMed, and Web of Science, which resulted in 123 reviewed papers. They performed an observation of patients with ASD who had increased total brain volume, especially less than six years old. Other significant differences reported in patients with ASD were volume throughout the frontal and temporal lobes, enlarged cortical thickness throughout the frontal lobe, enhanced cortical gyrification and surface area, and increased volume of cerebrospinal fluid. This reduces the cerebellum volume and reduces corpus callosum volume relative to typically developing controls. The results associated with the developmental progression of brain volume and age-related cortical thinning in ASD, along with possible volume variations in WM, basal ganglia, hippocampus, thalamus, and amygdala, were contradictory.

Lin et al. [43] identified most ASD vulnerability genes using machine learning methods from gene-level constraint metrics. The study applied features of spatiotemporal gene expression variations in the human brain and other gene variation characteristics. Genes found by their predictive model were enhanced with separate sets of ASD probability genes. They appeared to be distinguished in the brains of people with ASD, especially in the parietal and frontal cortex. Higher-ranking genes represented clear earlier proof of ASD participation and possibly unique candidates, like CAND1 (Cullin-Associated NEDD8-dissociated protein 1), MYCBP2 (MYC-Binding Protein 2), and DOCK3 (Dedicator of Cytokinesis 3), linked to neuronal growth.

In another investigation, Hegarty et al. [44] opined that Pivotal Response Treatment (PRT) was an efficient involvement of focusing on functional communication deficiencies and language delays, which were common and frequently observed in ASD. They investigated and suggested that neurobiological evaluation of language regions could give objective measures to help in the medical preparation of these deficiencies. 

Duda et al. [45] established a network known as brain tissue-specific Functional Relational Network (FRN), which applies machine learning techniques to predict the genomic-activated of autism concerned genes such as CTCF (11-zinc finger protein encoded gene), BRAF (serine and threonine kinase protein encoded gene), CHD7 (Chromodomain Helicase DNA 7), CHD8 (Chromodomain Helicase DNA 8), NTRK1 (Neurotrophic Tyrosine Receptor Kinase 1), and PTEN (Phosphatase and Tensin Homolog). This model recommends the number of unique genes with a high likelihood of contributing to ASD risk. Here, the authors utilized microarray data from GEO (Gene Expression Omnibus) for their analysis. 

The above analysis reveals various deficiencies such as language development, communication skills, social interactions, hyperactive, and repetitive behaviors to stimuli (e.g., taste, sound, light) are the most common symptoms in ASD. Several researchers have investigated the causes behind these symptoms. Specifically, the ASD individuals have unusual changes in brain structures such as increased volume in the whole brain and unusual genomic activations by various genes (top-rated genes or features) mentioned in the studies. 

### 7.5. Minimizing the Features That Contribute to Current ASD Methods without Impeding the Evaluation Metrics (Accuracy, Sensitivity, and Specificity)

The selection of features is an essential preprocessing step in data mining and machine learning studies. This process can reduce the amount of data analyzed and help build models with stronger interpretability based on optimal features. Pancerz et al. [46] introduced a preprocessing method for separating data objects. To clean training data from ASD, evaluation sheets are parts of the preprocessing operations to develop classifiers. Their approach divided the procedure into two steps: calculate the consistency factor and divide the set of all training cases into subsets of specific cases and boundary cases. This sheet consists of 70 cases where every subject was evaluated using questions grouped by 17 areas with 300 attributes. Every attribute had four values (0 = not performed; 25 = performed after receiving physical help; 50 = performed after receiving verbal help/demonstration; 100 = completed unaided).

Another characteristic of individuals with ASD has to do with recognizing and identifying vocal identity. In their article, Lin et al. [47] showed how persons with ASD recognize vocal identity. They exemplified three experiments in their study: gender discrimination, vocal identity recognition via naming and familiarity test.

In [19,26,48], the authors explained how to select the minimum features and classifications of ASD. The study by Hameed et al. [48] discussed gene expression in ASD. They downloaded autism microarray data from the National Center for Biotechnology Information’s Gene Expression Omnibus (GEO) public repository, which consists of 146 observations (samples) and 54,613 genes (features). The process was divided into three steps. In the first step, they checked similar gene expression characteristics in autism and control classes. They eliminated those genes whose mean and median were closest to >0.95. Through this process, they eliminated a significant number of genes (54,613 to 9454). In the second step, they applied three statistical filters: feature correlation (COR), the t-test (TT), and the Wilcoxon rank sum test (WRS). Finally, they applied a wrapper-based geometric binary particle swarm optimization support vector machine (GBPSO-SVM) algorithm to find the most discriminative genes with the presence of a repetitive gene (CAPS2), which was assigned as mostly related to ASD risk. The GBPSO-SVM algorithm improved the accuracy to 92.1%, whereas for Bi et al. [19], the classification accuracy was 96.15%. They applied a random SVM instead of a single SVM and used resting-state fMRI data acquired from ABIDE consisting of 46 typical controls and 61 individuals with autism. For the random SVM, the optimal feature achieved was 272 to differentiate people with autism and typical control individuals. However, they mentioned several restrictions, such as utilizing brain-level and not voxel-level features. As a feature, they simply applied four graph metrics, which were not many. Eventually, the performance of the random SVM depended on a single modal feature instead of multi-modal features. In their study, Wang et al. [26] applied support vector machine–recursive feature elimination (SVM-RFE), including a total of 255 subjects with autism and 276 subjects with typical development from 10 sites, to obtain optimal features based on functional connectivity (FC) and enhanced the classification accuracy on large-scale data. For global data sites, they achieved 90.60% accuracy (sensitivity 90.62%, specificity 90.58%), and for the leave-one-site-out test, the values were 75.00% to 95.23%.

The dependency and redundancy of features assessed individually by traditional feature selection approaches are the reasons for the inability to calculate their combined impact. For such cases, Teng et al. [49] applied an adaptive feature selection technique to evaluate the combined impact of different subsets on the entire feature space. This technique depended on V-shaped binary particle swarm optimization. 

Levy et al. [25] reduced features by detecting only a fundamental subset of behavioral features. In ADOS investigation, they efficiently categorized between ASD and non-ASD instances. In particular, developing a sparse system with a greater ability to generalize to the clinical community improved their preliminary study by taking score sheets of ADOS module 2 (ASD, 1319; non-ASD, 70) and module 3 (ASD, 2870; non-ASD, 273). They used datasets from Simons Variation in Individuals Project (Simons VIP), AGRE, SSC, and Boston AC for their investigation. The authors also employed 17 exceptional supervised learning approaches for 5 classification families.

Feature selection is an essential issue for the outstanding performance of model classification [7,8]. With seven features, Crippa et al. [50] were able to categorize the data of ASD children between age 2 to 4 years with upper-limb movements by applying supervised machine learning with SVM and achieved 96.7% accuracy. This study aimed to delve into the kinematic analysis of upper-limb movements and differentiate ASD from TD accurately. Though the result was promising, there were a few disadvantages, including limited data size, i.e., 15 ASD and 15 TD children. SVM had the best performance for a particular data sample, such as low-functioning and pre-schooling ASD children, and was inefficient for high-functioning ASD children, adults, and female patients.

Again, it is enough to discriminate between ASD and non-ASD individuals by taking only 10 features from 21 features of the datasets of autism, as Vaishali et al. [13] claimed. The authors recommended a single-objective binary firefly algorithm based on swarm intelligence for the selection of optimum features. They collected autism datasets from the University of California Irvine Machine Learning data Repository (UCI). 

Thabtah et al. [51] recommended a computational intelligence technique known as variable analysis (VA), which considerably decreases the sum of features for ASD screening techniques by retaining sensitivity, specificity, and analytical accuracy levels. They used datasets that were connected to AQ-Child, AQ-Adolescent, and AQ-Adult. VA can select a smaller number of features from the datasets compared to most other filtering techniques. By keeping satisfactory points of accuracy, sensitivity, and specificity, this technique decreased AQ-10 child, adolescent, and adult versions to 8, 8, and 6 features, respectively.

Finally, we have analyzed the various studies where the important issue is to optimize the features that directly affect ASD. So, the selection of essential features for ASD is conducted with the preprocessing step before diagnosing ASD. Therefore, researchers are very concerned about finding the minimum features. For example, seven features or ten features are considered a fundamental subset of behaviors to diagnose ASD. This issue’s main objective is that minimum features take minimum time (quick response); optimal features yield high accuracy.

## 8. Discussion

For ASD classification, maintaining accuracy, ensuring noise-free data, using a benchmark dataset, and reducing diagnostic time are essential issues. These significant issues depend on the exact feature selection of ASD because optimal features minimize data dimensionality, which reduces diagnostic time. 

In [10,12,36,37], the authors suggested using pre-diagnosis of ASD classification to minimize the diagnostic time. Allison et al. [36] fixed the cut-off point at six. In this point, for the AQ-10 child version, the sensitivity, specificity, and positive predictive value (PPV) were 0.95, 0.97, and 0.94, respectively; for the AQ-10 adolescent version, the values were 0.93, 0.95, and 0.86 respectively; and for the AQ-10 adult version, the values were 0.88, 0.91, and 0.85 respectively. Besides, for the Q-CHAT-10, the sensitivity, specificity, and PPV were 0.91, 0.89, and 0.58, respectively, when the cut-off point was three. However, on all measures, internal consistency was high, at greater than 0.85. Items were carefully chosen, which results in only the 10 best items for every instrument. The authors considered two samples for their research. One was a case sample consisting of more than 1000 participants with ASC (adults, 449; adolescents, 162; children, 432 and toddlers, 126) who were selected from the Autism Research Centre’s database of volunteers and a control sample consisting of 3000 individuals without ASC (adults, 838; adolescents, 475; children, 940 and toddlers, 754) from different sources. Participants finished full-length versions of the measures. 

In [10], though the authors showed behavioral differences between ASD and ADHD, which were time-sensitive, they selected important features for quick access to these risks. This study employed forward feature selection, under-sampling, and stratified 10-fold cross-validation to build a classifier. Among the six machine learning algorithms, four algorithms, logistic regression (LR), support vector classification (SVC), linear discriminant analysis (LDA), and categorical LASSO, chose only 5 out of 65 behaviors to differentiate autism from ADHD with an accuracy (AUC) of 0.965. Before this research, the authors also worked on diagnosing ASD risk using eight machine learning algorithms with backward feature selection from the score sheets of modules 2 and 3 from 4540 participants (3875 ASD, 665 non-ASD) [18]. They showed that the minimum features from module 2, 9 out of 28 behaviors, and module 3, 12 out of 28 actions, were good enough to identify ASD risk with accuracy 98.27% and 97.66%, respectively. They applied a fraction of the behaviors that are measured traditionally. Other researchers did the same analysis to identify further developmental delays. Duda et al. [10] extended their earlier work to analyze whether a limited set of behaviors obtained from the Social Responsiveness Scale (SRS) might be utilized to differentiate ASD from ADHD, thus allowing enhanced specificity when encountering clinically challenging cases. 

Another novel way to reduce ASD classification time is to use machine learning techniques with home videos of children proposed by Tariq et al. [37]. They claimed that their strategy reduced the classification time. In two different studies [12,37], they clarified that the current standard techniques for diagnosing ASD take a few hours to complete because they evaluate between 20 to 100 features. In [37], they employed eight machine learning algorithms with 162 home videos (within 2 min) for American children with and without ASD to measure the capability to detect ASD unfailingly on the mobile platform and attained 92% accuracy. In the subsequent study [12], the authors applied the new procedure to their model, like two classification layers. The first layer divided typical and atypical characteristics, and the second layer distinguished ASD and non-ASD. In this case, they also considered 159 home videos of Bangladeshi children on a mobile platform and achieved an accuracy of 76% in detecting atypical children from developmentally delayed children and 85% accuracy in recognizing children with ASD from those with other developmental delays. The authors proved that their technology could quickly determine ASD classification by tagging features of home videos of children in different cultures using machine learning on the mobile platform.

In summary, based on a critical review and analysis of recent investigations, this study demonstrated various uses of machine learning techniques in diagnosing and classifying ASD, along with their strengths and limitations. Critical issues, such as an imbalanced dataset, diagnosis time, classification accuracy, different evaluation measures, and essential feature selection and their techniques and datasets for rapid, accurate diagnosis of ASD, have been discussed and analyzed. However, certain issues still need to be addressed, such as learning from small amounts of data, using inappropriate sampling methods, and the dependency and redundancy of features by traditional feature selection approaches for combined impact problems that affect accuracy. The next research direction with machine learning in diagnosing ASD is to improve the diagnosis accurately, using less processing time with increasing complexity, without human interruption, a proper preprocessing plan, and methods to select best-ranked features—and, most importantly, to address data security issues using data sanitization methods.

## 9. Conclusions

In the study of ASD, diagnostic performance is vital and can be enhanced to classify the exact type of ASD accurately and cost-effectively. This can be accomplished in various ways, such as increasing predictive accuracy, maintaining sensitivity, specificity, and validity, and reducing diagnostic time. However, there has still been a deficiency in classification performance. Hence, further research is required for accurate and efficient classification of ASD. In this research direction, machine learning algorithms are applied as an intelligent method with minimal human involvement. Machine learning algorithms perform ASD diagnosis with encouraging outcomes. However, the vital issue with ASD diagnosis is performing efficient feature selection before implementing classification. Efficient feature selection can support accurate and effective classification. In this study, we show and discuss critical issues concerning autism, using machine learning algorithms. The emphasis is on selecting the optimal features of autism and improving classification while maintaining high accuracy. Therefore, by reducing data dimensionality and choosing the appropriate and essential autism features, a machine learning algorithm will show promising results in diagnosing ASD [13,18,25,52,53,54,55,56]. Nevertheless, several issues must be addressed, for instance, improper sampling methods, redundancy of features, imbalanced and insignificant data sizes, which influence accuracy. Future research with machine learning for diagnosing ASD is to enhance the predictive model’s performance accurately, minimize those critical issues without human interruption and, very importantly, address the security problems of ASD datasets by applying data sanitization techniques.

## Figures and Tables

**Table 1:** Criteria for inclusion and exclusion.

Criteria for Inclusion	Criteria for Exclusion
Articles in English language (AND)	Articles unrelated to ASD and similar disorders (AND)
Studies that are peer-reviewed (AND)	Papers that not related autism dataset (AND)
Articles published mostly in 2015 to 2020 (AND)	Studies that isolated autism with machine learning (AND)
Papers related to autism dataset (AND)	Articles that did not fulfill any criterion of inclusion
Articles emphasized classification and feature selection of ASD (AND)	
Articles that analyzed ASD and other similar disorders (AND)	
Studies related to autism with machine learning (AND)	
Articles that related to ASD datasets with users’ security and privacy	

**Table 2:** Various sources of autism datasets.

Source No.	Data Source	Website
01	UCI	https://archive.ics.uci.edu/ml/datasets.php
02	ABIDE I	http://fcon_1000.projects.nitrc.org/indi/abide/abide_I.html
03	ABIDE II	http://fcon_1000.projects.nitrc.org/indi/abide/abide_II.html
04	NDAR	https://ndar.nih.gov
05	AGRE	https://www.autismspeaks.org/agre
06	NRGR	https://www.nimhgenetics.org
07	GEO	https://www.ncbi.nlm.nih.gov/geo
08	SSC	https://www.sfari.org/resource/simons-simplex-collection
09	Simons VIP	https://www.sfari.org/funded-project/simons-variation-in-individuals-project-simons-vip

UCI, University of California Irvine Machine Learning Repository; ABIDE I and II, Autism Brain Imaging Data Exchange I and II; NDAR, National Database for Autism Research; AGRE, Autism Genetic Resource Exchange; NRGR, National Institute of Mental Health Repository and Genomics Resource; GEO, Gene Expression Omnibus; SSC, Simons Simplex Collection; Simons VIP, Simons Variation in Individuals Project.

**Table 3:** Summary of relevant works that utilized machine learning algorithms for the classification of ASD.

Authors/ Year/Reference	Data Type	Sample Data Size	Algorithms/ Methods	Predictive Objectives	Accuracy	Sensitivity	Specificity
Duda et al. (2016) [10]	SRS	2775 ASD, 150 ADHD	LASSO, SVM, LDA, Ridge regression	ASD/ADHD	96.5%	-	-
Bi et al. (2018) [19]	rs-fMRI	61 ASD, 46 TC	Random SVM	ASD/TC	96.15%	-	-
Heinsfeld et al. (2018) [20]	rs-fMRI	505 ASD, 530 TC	Deep learning	ASD/TC	70%	74%	63%
Duda et al. (2016) [21]	ADI-R	891 ASD, 75 non-ASD	ADTree	ASD/non-ASD	-	89.9%	79.7%
Bone et al. (2016) [17]	ADI-R, SRS	1264 ASD, 462 other DD	SVM	ASD/other DD	-	(87%, 89%)	(53%, 59%)
Kong et al. (2019) [22]	T1-wMRI	78 ASD, 104 TC	Deep learning	ASD/TC	90.39%	84.37%,	95.88%
Kosmicki et al. (2015) [18]	ADOS-2	3885 ASD, 665 non-ASD	ADTree, SVM, Ridge regression	ASD/non-ASD	(98%, 98%)	(99%, 98%)	(89%, 97%)
Duda et al. (2017) [23]	SRS	3023 ASD, 324 ADHD	ENet, LASSO, SVM, LDA, Ridge regression	ASD/ADHD	90%	-	-
Achenie et al. (2019) [24]	M-CHAT-R/F	14,995 Toddlers	fNN	ASD/non-ASD	(99.64%, 99.95%)	73.8%	99.9%
Levy et al. (2017) [25]	ADOS	1319 ASD, 70 non-ASD & 2870 ASD, 273 non-ASD	Supervised learning	ASD/non-ASD	-	-	-
Vaishali et al. (2018) [13]	ADOS, ADI-R	292 ASD	Binary firefly algorithm	ASD/non-ASD	92.12–97.95%	-	-
Wang et al. (2020) [26]	rs-fMRI	255 ASD, 276 TD	SVM-RFECV	ASD/TD	(90.60%, 75–95.23%)	90.62%	90.58%

SRS, Social Responsiveness Scale; rs-fMRI, resting state-functional Magnetic Resonance Imaging; T1-wMRI, T1-weighted Magnetic Resonance Imaging; ADOS-2, Autism Diagnostic Observation Schedule-2; ADI-R, Autism Diagnostic Interview-Revised; M-CHAT-R/F, Modified Checklist for Autism in Toddlers-Revised/Follow-Up; DD, Developmental Disorders; TD, Typical Development; LASSO, Least Absolute Shrinkage and Selection Operator; SVM, Support Vector Machine; LDA, Linear Discriminant Analysis; ADHD, Attention Deficit Hyperactivity Disorder; TC, Typical Control; ADTree, Alternating Decision Tree; ENet, Elastic Net; fNN, feedforward Neural Network; SVM-RFECV, Support Vector Machine-Recursive Feature Elimination with a stratified-4-fold Cross-Validation.
